# Measurement of ground reaction forces in cats after total hip replacement

**DOI:** 10.1177/1098612X241297894

**Published:** 2024-12-20

**Authors:** Gregor Schweng, Barbara Bockstahler, Alexander Tichy, Klaus Zahn, Georg Haimel, Günter Schwarz, Eva Schnabl-Feichter

**Affiliations:** 1 Department for Companion Animals and Horses, University Clinic for Small Animals, Small Animal Surgery, University of Veterinary Medicine, Vienna, Austria; 2 Department for Companion Animals and Horses, University Clinic for Small Animals, Small Animal Surgery, Section for Physical Therapy, University of Veterinary Medicine, Vienna, Austria; 3 Department for Biomedical Science, Platform Bioinformatics and Biostatistics, University of Veterinary Medicine, Vienna, Austria; 4 Small Animal Clinic Ismaning, Ismaning, Germany; 5 Tierarztpraxis am Stadtpark, Vienna, Austria; 6 Anicura Small Animal Clinic Hollabrunn, Hollabrunn, Austria

**Keywords:** Total hip replacement, hip arthroplasty, gait analysis, ground reaction forces, pressure-sensitive plate, femoral head ostectomy

## Abstract

**Objectives:**

The aim of this study was to evaluate ground reaction forces (GRFs) in cats after unilateral total hip replacement (THR) and compare them with cats after femoral head and neck ostectomy (FHO).

**Methods:**

The databases of the Small Animal Clinic of the Veterinary University in Vienna and three referral clinics were searched for cats that had undergone unilateral THR with the BioMedtrix Micro total hip system or FHO more than 6 months previously. Owners were invited to complete a survey and bring their cats for re-examination, inlcuding clinical and orthopaedic examinations, hip radiography and a gait analysis using a pressure-sensitive plate.

**Results:**

Nine cats were included in each group. Cats after THR showed larger GRF values (peak vertical force [PFz] and vertical impulse [IFz] normalised to total force [%TF]) on the operated limb. The resulting symmetry indices (SIs) were lower in terms of vertical force in 7/9 (78%) cats and vertical impulse in 6/9 (67%) cats between the hindlimbs in cats after THR compared with FHO – SI (PFz) = 3.31% ± 2.19% (THR) vs 4.84% ± 2.99% (FHO) and SI (IFz) = 5.17% ± 3.66% (THR) vs 8.27% ± 3.12% (FHO). Cats after FHO showed significantly lower muscle circumference and range of motion (ROM) at the operated hindlimb compared with the contralateral side, whereas cats after THR showed no statistically significant differences between their hindlimbs. Owner surveys revealed significant differences in their subjective assessment of activity and change in gait between the two groups, with better values for cats after THR.

**Conclusions and relevance:**

This was the first study that measured GRFs in cats after THR. PFz (%TF) and IFz (%TF) values were higher in the operated limb of the THR group than in those of the FHO group, resulting in lower symmetry indices (indicating better symmetry) and better loading of the corresponding hindlimb. This finding is clinically relevant and can help in making decisions regarding the treatment of hip joint pathologies in cats.

## Introduction

Injuries and pathologies of the hip joint in cats are common and primarily include fractures, luxations and hip dysplasia with consecutive osteoarthritis.^1–9^

Total hip replacement (THR) is the gold standard of therapy in dogs, whereas femoral head and neck ostectomy (FHO) is still commonly performed in cats. This surgical technique provides good outcomes;^10–12^ however, gait analyses revealed significant differences between the visual perception of the gait pattern and the actual objective measurement using pressure-sensitive plates, 1 year after FHO.^13^

Currently available THR systems for cats are the cemented BioMedtrix (CFX) Micro and Nano Hip systems and the Zurich Mini Cementless Hip Replacement System (Kyon). BioMedtrix press-fit cups (BFX) have also recently become available.^14^ Studies have reported success in performing THR in cats; however, these were based solely on findings from orthopaedic and radiological examinations and owner surveys.^15–23^

The measurement of ground reaction forces (GRFs) using a pressure-sensitive walkway represents a suitable method for the objective determination of lameness and therapy success.^24–28^ To date, studies have published results of gait analysis after FHO in cats^11,13^ but not after THR. Therefore, the aim of this study was to evaluate gait patterns of cats after THR on a pressure-sensitive plate and compare them with findings after FHO. Thus, we hypothesised that cats that had undergone THR objectively show equal loading (symmetry) of the hindlimbs, that more than 50% of the examined cats demonstrate better weightbearing (therefore lower symmetry indices) than after FHO, and that clinical and radiological findings correlate with biomechanical changes that can be visualised using a pressure-sensitive plate.

## Materials and methods

### Animals

This study was approved by the Institutional Ethics and Animal Welfare Committee, in accordance with the guidelines for Good Scientific Practice and national legislation (reference numbers ETK-003/01/2022 and ETK-104/06/2023). The databases of the Small Animal Clinic of the Veterinary University in Vienna and three referral clinics (Small Animal Clinic Ismaning, Germany; Tierarztpraxis am Stadtpark, Austria; and Anicura Small Animal Clinic Hollabrunn, Austria) were searched for cats that had undergone unilateral THR or FHO more than 6 months previously. THR was performed using the BioMedtrix Micro Cemented Fixation (CFX) system (#3 Stem; 12, 14 or 16 mm cup and 8 mm head +0 or +2 neck). The exclusion criteria included bilateral THR or FHO, cats that had surgery less than 6 months previously, patients with other orthopaedic conditions on the same or contralateral leg with clinical manifestation or corresponding surgeries, or cats for which a complete re-examination was not possible.

### Study design

Owners were invited to being their cats for an orthopaedic re-examination and gait analysis. Clinical and radiological examinations and GRF measurements were conducted at the University of Veterinary Medicine Vienna or the Small Animal Clinic Ismaning.

The cat owners received a questionnaire in German (adapted from UNESP-Botucatu Multidimensional Composite Pain Scale^29^), which was also used in a previous study about GRF measurement in cats after FHO.^13^ This questionnaire contained 11 questions with a maximum score of 110 points (see File 1 in the supplementary material).

The gait analysis was performed using a pressure measurement plate (Zebris FDM Type 2; Zebris Medical; see File 2 in the supplementary material), which was placed in the middle of a quiet room. After a short acclimatisation phase, the cats were motivated to walk lengthwise over the pressure plate with the help of verbal and visual stimuli, food and/or toys. The measurement was rated, if at least five valid step cycles could be measured. Gait cycles were excluded when the cat stopped or had an apparent change in velocity, turned its head or left the plate. Cats that did not cross the plate voluntarily were excluded from the study. All measurements were video recorded so that the legs could be allocated accordingly.

After a brief general clinical examination, a complete orthopaedic examination was performed. This included a visual lameness examination, palpation, range of motion (ROM) measurement using goniometry and recording of muscle circumference at the midpoint of the thigh using a tape measure.

Concluding the re-examination, radiographs of the entire pelvis and hip joint in ventrodorsal and laterolateral beam paths were taken without sedation.

### Data processing and parameters

All collected data were processed using a specially developed software (Pressure Analyzer 1.3.0.2; Michael Schwanda).

Gait velocity (in m/s) was calculated from the left forelimb. Parameters that were evaluated included PFz (N) and IFz (Ns), which were normalised to total force (%TF).^30^ The symmetry index (SI [%]) values for the forelimbs and hindlimbs were calculated from PFz (SI[PFz]) and IFz (SI[IFz]), as described previously (see File 3 in the supplementary material);^30–32^ therefore, an SI of 0% would represent perfect symmetry between the contralateral limb pair. For easier comparison between groups, the extremities were named based on the operated limb, hindlimb THR or FHO (HL-THR/FHO), hindlimb contralateral (HL-CL), forelimb ipsilateral (FL-IPS) and forelimb contralateral (FL-CL), regardless of whether the THR or FHO was performed on the left or right side. In addition, other temporospatial parameters, such as step length (SL [m]), paw contact area (PCA [cm^2^]), stance phase duration (SPD [s]) and time to maximum load in %SPD (TPFz) were determined.

### Statistical analysis

All measured data were processed using SPSS statistical software version 24 (IBM). The Shapiro–Wilk test was used to determine the normal distribution of the data. Data are presented as mean ± SD. A general linear model was used to compare the limbs within and between the groups. To detect significant differences between groups in the individual parameters, independent *t*-tests were applied. Using Pearson’s correlation coefficient, results of the orthopaedic examination and values from the owner’s survey were correlated with GRFs and temporospatial parameters. All presented data were normally distributed. For all statistical analyses, *P* <0.05 was considered significant.

## Results

**Figure 1:**
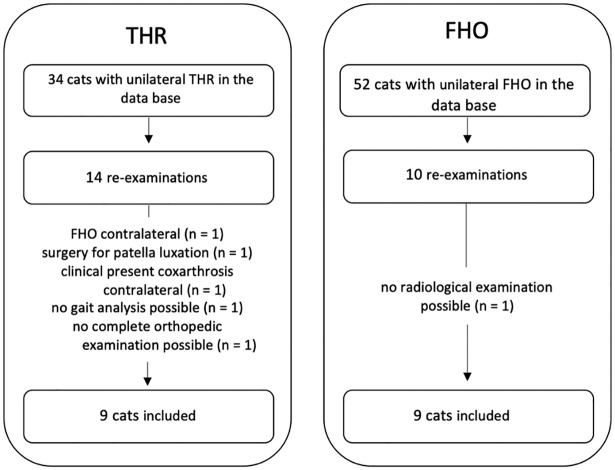
Flowchart of the chronology of acquiring cats for re-examinations. FHO = femoral head and neck ostectomy; THR = total hip replacement

After implementation of the exclusion criteria, nine cats in both groups could be included (Figure 1). The THR group included four Maine Coon cats, two domestic shorthair cats, two British Shorthairs and one Bengal. The FHO group included five Maine Coon cats, one Ragdoll, one Bengal, one British Shorthair and one domestic shorthair cat. The mean body weight was 6.4 ± 2.1 kg (range 4.3–9.6) in the THR group and 5.9 ± 1.9 kg (range 4.0–10.0) in the FHO group, with no significant difference between the two groups. There were seven castrated males in the THR and FHO group, two spayed females in the THR group, one in the FHO group and one intact female in the FHO group.

The mean age at the time of surgery was 2.9 ± 2.3 years (range 1.2–8.2) in the THR group and 1.8 ± 0.9 years (range 0.4–3.7) in the FHO group, with no significant difference. At the time of re-examination, the mean age was 7.3 ± 2.1 years (range 4.8–11.5) in the THR group and 3.7 ± 1.8 years (range 2.3–7.3) in the FHO group. The mean interval between surgery and re-examination was significantly longer (*P* <0.01) in the THR group (4.4 ± 1.4 years; range 3.1–7.1) than in the FHO group (1.9 ± 1.1 years; range 0.8–4.1). The different time intervals between the two groups had no significant influence on the presented results.

THRs were performed for slipped capital physis and hip dislocation in three cats each and femoral head and neck fracture, coxarthrosis and combined hip dislocation with femoral head fracture in one cat each. FHOs were performed for femoral head and neck fracture in four cats, hip luxation in three and slipped capital physis in two.

### Orthopaedic examination

In the orthopaedic examination, 7/9 cats in the THR group were lameness free (grade 0/5) and 2/9 cats had grade 1/5 lameness at the corresponding hindlimb. In the FHO group, 1/9 cats were categorised as grade 0/5, 3/9 as grade 1/5, 4/9 as grade 2/5 and 1/9 as grade 3/5 lameness. On palpation, 6/9 cats in the FHO group exhibited pain during hip manipulation, whereas none of the THR cats did. Although not significant, cats in the FHO group with a higher body mass also showed a higher lameness score (*r* = 0.53, *P* = 0.15).

The FHO group showed a significant reduction in ROM (*P* <0.01) and muscle circumference (*P* <0.01) on the operated limb compared with the non-operated hindlimb. Hip extension was significantly reduced (*P* = 0.04) in the FHO group compared with the THR group at the operated limb (Table 1).

**Table 1:** Orthopaedic examination: comparison of ROM and muscle circumference between the THR and FHO groups

Variable	THR	FHO
Total ROM (°)		
HL-THR/FHO	119.78 ± 7.77 (110–130)	101.56 ± 14.24 (78–122)*
HL-CL	120.89 ± 9.28 (108–134)	118.44 ± 12.68 (90–130)*
Flexion (°)		
HL-THR/FHO	39.33 ± 8.00 (30–52)	45.33 ± 5.39 (36–54)
HL-CL	39.33 ± 8.83 (30–56)	38.00 ± 9.59 (28–54)
Extension (°)		
HL-THR/FHO^†^	159.11 ± 6.09 (150–168)	146.89 ± 11.01 (132–162)
HL-CL	160.22 ± 6.12 (146–166)	156.44 ± 9.53 (140–172)
Muscle circumference (mm)		
HL-THR/FHO	208.00 ± 42.57 (160–288)	193.89 ± 24.02 (160–225)*
HL-CL	211.67 ± 38.67 (165–285)	212.33 ± 25.58 (172–248)*

Data are mean ± SD (range)

* Significant difference compared with contralateral hindlimb within the group

† Significant difference between the THR and FHO groups

FHO = femoral head and neck ostectomy; HL-CL = contralateral hindlimb; HL-THR/FHO = operated hindlimb; ROM = range of motion; THR = total hip replacement

### Radiological findings

Radiological re-examination of the THR group showed osteophytic formations cranial of the cup in 4/9 cats and osteophytic formations at the osteotomy side, as well as periosteal reactions at the metaphysis and proximal diaphysis of the femur in 3/9 cats. Four cats exhibited signs of hip joint dysplasia with consecutive coxarthrosis of the contralateral hip, without clinical manifestations. One cat exhibited lateral displacement of the hip stem within the medullary canal with consecutive axial malalignment of the femur in sense of coxa vara, resulting in increased SI(PFz) and SI(IFz) values but without clinically perceptible lameness. The two cats that showed lameness of 1/5 on the operated limb had radiological signs of coxarthrosis on the contralateral side, but no changes in the area of the operated limb. In the FHO group, 2/9 cats displayed a dorsally displaced trochanter, 3/9 cats exhibited osteophytic formations at the acetabulum, 7/9 cats presented osteophytic formations in the area of the osteotomy site and 4/9 showed signs of hip joint dysplasia on the contralateral side; however, without manifestation in the orthopaedic examination. For the two cats with dorsal trochanter displacement, one had increased SI(PFz) and SI(IFz) values of the hindlimbs, whereas the other did not.

### GRF measurement

Mean gait velocity of the left forelimb was 0.58 ± 0.09 m/s (range 0.47–0.77) in the THR group and 0.66 ± 0.08 m/s (range 0.51–0.79) in the FHO group.

**Figure 2:**
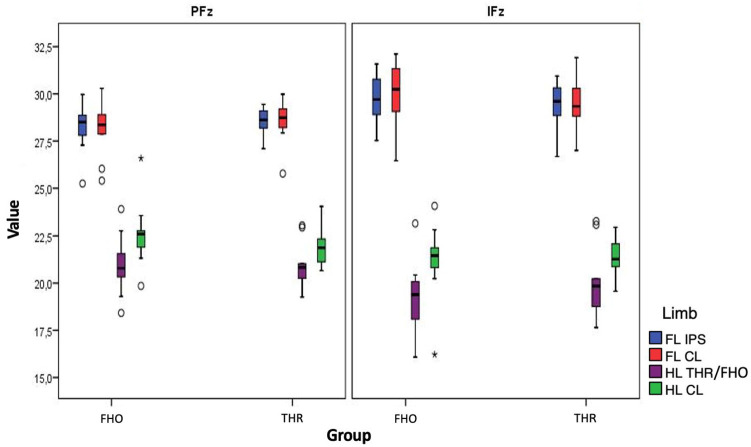
GRFs (peak vertical force [PFz] and vertical impulse [IFz] normalised to total force [%TF]) of the FHO and THR groups. The solid line within the box represents the median, the lower and upper limits of the box represent the interquartile (25th and 75th percentiles) range, respectively; the whiskers delimit the range. Values observed more than 1.5 times of the IQR below the first quartile (Q1) or above the third quartile (Q3) are marked as a circle. Values more than three times the IQR below Q1 or above Q3 are marked with an asterisk. FHO = femoral head and neck ostectomy; FL-CL = contralateral forelimb; FL-IPS = ipsilateral forelimb; GRF = ground reaction force; HL-CL = contralateral; HL-THR/FHO = operated hindlimb; IQR = interquartile range; THR = total hip replacement

Mean PFz(%TF) and IFz(%TF) values on the operated hindlimbs were 20.98 ± 1.29 and 20.01 ± 2.01 in the THR group and 20.97 ± 1.67 and 19.16 ± 2.08 in the FHO group, respectively (Table 2). The values of the corresponding contralateral hindlimbs were 22.01 ± 1.02 and 21.30 ± 1.18 in the THR group and 22.62 ± 1.83 and 21.13 ± 2.16 in the FHO group, respectively (Figure 2). The resulting SI(PFZ) of the hindlimbs and forelimbs in the THR group was 3.31% ± 2.19% and 0.86% ± 0.80%, respectively, and their SI(IFz) was 5.17% ± 3.66% and 2.07% ± 2.05%, respectively. Corresponding SIs in the FHO group were 4.84% ± 2.99% and 0.91% ± 0.74%, respectively, and 8.27% ± 3.12% and 1.84% ± 1.09%, respectively. Thus, the THR group showed lower SIs in terms of vertical force in 7/9 (78%) cats and vertical impulse in 6/9 (67%) cats between the hindlimbs, although these were not significant (SI(PFz), *P* = 0.24; SI(IFz), *P* = 0.07). In both groups, the SI(PFz) and SI(IFz) of the forelimbs were significantly lower than those of the corresponding hindlimbs (THR group, *P* <0.01 and *P* = 0.04; FHO group, *P* <0.01 and *P* <0.01, respectively). Cats in the THR group that had increased SI(PFz) values also showed a significantly reduced muscle circumference at the operated hindlimb (*r* = −0.80, *P* <0.01). A significant correlation was found between increasing body mass and increasing SI(PFz) of the forelimbs after excision arthroplasty (*r* = 0.69, *P* = 0.04).

**Table 2:** Ground reaction forces (normalised to the total force [%TF]) of cats after unilateral THR or FHO

Limb	PFz (%TF)	IFz (%TF)
FL-IPS		
THR	28.51 ± 0.71 (27.10–29.44)	29.32 ± 1.37 (26.68–30.94)
FHO	28.25 ± 1.40 (25.25–29.97)	29.66 ± 1.36 (27.53–31.58)
FL-CL		
THR	28.51 ± 1.20 (25.78–29.98)	29.38 ± 1.58 (27.00–31.92)
FHO	28.16 ± 1.58 (25.40–30.29)	30.05 ± 1.86 (26.46–32.11)
HL-THR/FHO		
THR	20.98 ± 1.29 (19.26–23.06)*^†^	20.01 ± 2.01 (17.65–23.28)*^†^
FHO	20.97 ± 1.67 (18.42–23.92)*^†^	19.16 ± 2.08 (16.08–23.16)*^†^
HL-CL		
THR	22.00 ± 1.02 (20.66–24.06)	21.30 ± 1.18 (19.57–22.96)
FHO	22.62 ± 1.83 (19.85–26.59)	21.13 ± 2.16 (16.22–24.09)

Data are mean ± SD (range)

* Significant difference compared with ipsilateral forelimb

† Significant difference compared with contralateral forelimb

FHO = femoral head and neck ostectomy; FL-CL = forelimb contralateral; FL-IPS = forelimb ipsilateral; HL-CL = hindlimb contralateral; HL-IFz = vertical impulse; HL-THR/FHO = hindlimb with total hip replacement or FHO; PFz = peak vertical force; ROM = range of motion; THR = total hip replacement

A significant correlation was found between decreasing PFz(%TF) of the operated hindlimb and increasing PFz(%TF) and IFz(%TF) for both forelimbs in the FHO group [PFz(%TF) FL-IPS, *P* <0.01; FL-CL, *P* <0.05; IFz(%TF) both forelimbs, *P* <0.05] and the contralateral forelimb in the THR group (*P* <0.01 each).

A correlation was noted between increasing SI(IFz) of the hindlimbs and increasing IFz(%TF) of the contralateral forelimb in both groups, but only significantly in the FHO group (*r* = 0.72, *P* = 0.03). In the FHO group, a correlation was observed between painful hip extension during orthopaedic examination and reduced IFz(%TF) values on the operated hindlimb (*P* <0.05).

In both groups, the TPFz(%SPD) was significant later in the forelimbs (*P* <0.01 for both groups) than in the hindlimbs. No significant differences in TPFz(%SPD) were noted between the forelimbs of both groups. However, the THR group showed significance later TPFz(%SPD; *P* <0.05) on the operated limb than the FHO group.

### Temporospatial parameters

The THR group showed significant longer SPD (*P* <0.03) for all four limbs compared with the FHO group. Within the group, cats after FHO showed shorter SPD of the operated hindlimb compared with the three remaining legs (HL-CL, *P* = 0.48; FL-IPS, *P* <0.01; FL-CL, *P* = 0.01). No significant differences in SL and PCA were found between the limbs within the groups and between the groups (Table 3).

**Table 3:** Temporospatial parameters of cats after unilateral THR or FHO

Limb	TPFz (%SPD)	SPD (s)	SL (m)	PCA (cm^2^)
FL-IPS				
THR	47.79 ± 5.20 (39.41–56.65)	0.63 ± 0.08 (0.45–0.72)*	0.53 ± 0.06 (0.47–0.64)	13.08 ± 4.73 (8.37–21.09)
FHO	53.87 ± 10.53 (31.50–67.78)	0.54 ± 0.061 (0.45–0.63)*	0.53 ± 0.05 (0.44–0.59)	15.99 ± 2.04 (12.10–18.57)
FL-CL				
THR	50.13 ± 6.38 (35.97–58.32)	0.63 ± 0.08 (0.46–0.72)*	0.52 ± 0.06 (0.46–0.61)	13.27 ± 5.08 (7.77–22.83)
FHO	56.31 ± 10.01 (34.40–67.52)	0.54 ± 0.07 (0.46–0.62)*	0.53 ± 0.05 (0.44–0.60)	16.14 ± 1.99 (12.10–18.33)
HL-THR/FHO				
THR	35.09 ± 5.02 (27.44–41.86)*^† ‡^	0.58 ± 0.07 (0.46–0.71)*^†^	0.54 ± 0.06 (0.45–0.63)	12.72 ± 5.03 (7.65–22.66)
FHO	28.16 ± 8.32 (21.18–46.97)*^† ‡^	0.48 ± 0.06 (0.39–0.57)*^† ‡^	0.54 ± 0.04 (0.46–0.59)	15.24 ± 2.65 (10.85–18.95)
HL-CL				
THR	34.33 ± 8.94 (25.71–51.71)	0.59 ± 0.07 (0.44–0.68)*	0.54 ± 0.07 (0.44–0.65)	12.87 ± 4.40 (7.65–19.96)
FHO	28.09 ± 8.62 (19.35–48.04)	0.51 ± 0.07 (0.43–0.62)*	0.53 ± 0.04 (0.45–0.59)	15.81 ± 2.54 (11.12–20.08)

Data are mean ± SD (range)

* Significant difference between THR and FHO cats

† Significant difference compared with ipsilateral forelimb

‡ Significant difference compared with contralateral forelimb

FHO = femoral head and neck ostectomy; FL CL = forelimb contralateral; FL IPS = forelimb ipsilateral; HL CL = hindlimb contralateral; HL THR/FHO = hindlimb with total hip replacement or FHO; PCA = paw contact area; SL = step length; SPD = stance phase duration; THR = total hip replacement; TPFz = time to PFz (peak vertical force)

### Owner survey

The owner survey recorded a mean of 21 ± 7 points (range 11–31) in the THR group and 35 ± 16 points (11–55) in the FHO group, showing significant differences (*P* <0.04) (Table 4). A significant correlation was identified between an increased SI(PFz) of the hindlimbs and a poorer evaluation by the owner in the categories jumping on elevations (*r* = 0.6, *P* <0.01), jumping from elevations to the ground (*r* = 0.61, *P* <0.01) and change in gait pattern at the present time (*r* = 0.53, *P* <0.05).

**Table 4:** Results of the owner survey for each question presented as a comparison of the total hip replacement (THR) group and the femoral head and neck ostectomy (FHO) group

Question	THR	FHO
Total value	21 ± 7 (11–31)	35 ± 16 (11–55)
General behaviour	3 ± 2 (1–7)	3 ± 2 (1–7)
General activity of the cat	3 ± 2 (1–5)	3 ± 2 (1–7)
Running*	1 ± 1 (1–3)	3 ± 2 (1–6)
Running after lying for a while	2 ± 1 (1–4)	3 ± 2 (1–7)
Jumping on elevation*	2 ± 2 (1–5)	5 ± 3 (1–8)
Jumping from elevation to the ground*	2 ± 1 (1–4)	4 ± 3 (1–8)
Climbing stairs	2 ± 2 (1–7)	3 ± 2 (1–5)
Playing with toys	2 ± 2 (1–5)	2 ± 1 (1–4)
Pain	1 ± 1 (1–2)	3 ± 2 (1–6)
Gait change at the present*	1 ± 0 (1–2)	4 ± 2 (1–8)
Appetite	2 ± 1 (1–5)	2 ± 1 (1–5)

Data are mean ± SD (range)

* Significant difference between the THR and FHO groups

FHO = femoral head and neck ostectomy; THR = total hip replacement

Further results and findings can be found in Files 4 and 5 in the supplementary material.

## Discussion

This study proved the hypothesis that cats after THR showed better loading of the operated hindlimb than cats after FHO, resulting in lower SIs (better symmetry) in more than 50% of the examined cats. In general, correlations between the results of the orthopaedic examination and the results of the gait analysis can be identified; however, not all cats that have shown altered GRF values have been diagnosed with lameness during the orthopaedic examination. These findings correlate with previous results after GRF measurements in cats after FHO.^13^ Nevertheless, owners were consistently able to assess and rate their cats accurately.

THR is a salvage procedure to eliminate the source of pain and restore the function of the hip joint. Since the introduction of micro and nano THR systems, this procedure is also available for small dogs and cats.^14,16,17^ We compared two salvage procedures and revealed better clinical outcomes in cats after THR. Thus, this group showed less lameness and no difference in ROM between the operated and healthy limbs, whereas cats after FHO showed marked clinical gait changes and significantly reduced ROM on the operated limb. In the present study, none of the cats in the THR group showed pain with passive hip movements and no significant differences in hindlimb muscle circumference, whereas the majority of cats in the FHO group did, which is a sign of unloading of the corresponding extremity. These findings correlated with previously reported outcomes after FHO.^12,13,15^

Radiological findings in the present study are consistent with previously described findings for THR or FHO.^12,13,16–18^ In the present study, one cat showed axial malalignment of the stem causing coxa vara, which was also described previously.^16,17^ This cat showed no clinical lameness but increased SIs of the hindlimbs. Whether this malalignment is responsible for the increased SI values remains unclear, which may be through another load distribution within the leg. In this study, 8/9 cats in the FHO group showed exostosis in the area of the femoral ostectomy site, the acetabulum or both. This finding is consistent with previous findings after FHO.^12,13^ Off and Matis revealed that bony proliferations which developed at the resection site or the lesser trochanter did not correlate with functional outcome.^12^ In fact, as nearly all cats in our FHO group presented with exostosis, and we were unable to compare them with a group without these bony proliferations, the effect of gait abnormalities caused by osteophytic formations should be further investigated.

Although the difference was not significant, the THR group showed higher GRF values on the operated hindlimb than the FHO group; therefore, SIs were lower in terms of the vertical force in 7/9 cats and impulse in 6/9 cats between the hindlimbs in cats after THR compared with FHO. Thus, cats walked less lame after THR and therefore put more strain on the operated limb. Cats after excision arthroplasty showed shorter SPD values on the operated hindlimb than on the other ones. This result is similar to earlier findings, where GRFs were measured after FHO or stifle pathologies in cats.^13,30^

In this study, correlations were found between changing GRF values of the operated hindlimb and those of the other limbs, especially the contralateral forelimb. This seems to be a compensatory mechanism, where the body weight is shifted to the remaining limbs, with a tendency towards the thoracic limbs. Earlier findings regarding the compensatory mechanism showed that cats redistributed these forces equally to all other three limbs.^28^ In another study, cats tended to compensate hindlimb lameness by shifting weight to their forelimbs, especially to the contralateral one, which is consistent with our results.^13^ Similar compensatory mechanisms have been described in dogs.^33,34^

Previously, GRF measurements were used to evaluate the outcome 1 year after FHO.^13^ Comparing these results with the findings of the present study, the THR group showed lower SIs of the hindlimbs. Cats in the FHO group showed higher mean SIs than these previously published values of SI(PFz) (3.54% ± 1.73%) and SI(IFz) (7.17% ± 3.64%).^13^ Comparing the two FHO groups, Schnabl-Feichter et al^13^ investigated only domestic shorthair cats with a mean weight of 4.6 ± 1.2 kg. Conversely, our group enrolled several breeds, with an overrepresentation of Maine Coon cats and a mean weight of 5.9 ± 1.9 kg. In the present study, the FHO group showed a mild correlation between increasing body weight and increasing lameness scores in the orthopaedic examination. Although these results were not significant, earlier studies have shown poorer results with increasing weight after FHO, particularly in dogs.^12,35^ Further investigation is needed to prove the influence of body weight on the outcome after FHO and THR in cats.

Owner surveys expressed very good satisfaction with the outcome of both surgical techniques. This is consistent with the findings of previous studies.^10,12,13,15,16,18^ Nevertheless, in the present study, the THR group achieved significantly better owner satisfaction than the FHO group. Despite the subjective influence of a clinically inexperienced owner, this study shows that cats that performed worse in the orthopaedic examination and gait analysis were also rated poorly by the owner. These observations must be viewed with caution but show that owners can correctly perceive and assess these changes and can therefore provide important assistance in the diagnosis of orthopaedic conditions.

The limitations of this study are primarily its retrospective nature, because all cats were operated at least 6 months previously and the time between surgery and control examination varied greatly. Furthermore, the findings are limited by the small number of cats in both groups, owing to the currently still small number of cats that have undergone unilateral THR. A further limitation is that there was only one GRF measurement and examination at a previously undefined time postoperatively; therefore, it is not clear whether the results would have been different at another time point or if they would have varied over time. The subjective influence of the owner surveys also limited the interpretation of the results; thus, comparison should be made with caution.

## Conclusions

To the best of the authors’ knowledge, this is the first study that measured GRFs in cats after THR. PFz(%TF) and IFz(%TF) values were higher in the operated limb of cats after THR compared with FHO, resulting in lower SIs and better loading of the corresponding hindlimb. The results of the orthopaedic examination and owner survey also correlated with GRFs and temporospatial parameters. Conclusions should be drawn with caution owing to the small number of unilateral THR cases in cats; thus, further investigations are needed to prove the presented results. Nevertheless, the findings are clinically relevant and should help in making decisions regarding the treatment of hip joint pathologies in cats.

## Supplemental Material

sj-docx-2-jfm-10.1177_1098612X241297894 – Supplemental material for Measurement of ground reaction forces in cats after total hip replacement File 2: Technical data of the pressure-sensitive plate.

sj-docx-3-jfm-10.1177_1098612X241297894 – Supplemental material for Measurement of ground reaction forces in cats after total hip replacement File 3: Calculation of symmetry index.

sj-docx-4-jfm-10.1177_1098612X241297894 – Supplemental material for Measurement of ground reaction forces in cats after total hip replacement File 4: Additional results and findings.

sj-docx-5-jfm-10.1177_1098612X241297894 – Supplemental material for Measurement of ground reaction forces in cats after total hip replacement File 5: Lameness scores.

sj-pdf-1-jfm-10.1177_1098612X241297894 – Supplemental material for Measurement of ground reaction forces in cats after total hip replacement File 1: Owner survey.
