# Supplementary material for: Measurement of ground reaction forces in cats after total hip replacement
Source: J Feline Med Surg. 2024 Dec 20;26(12):1098612X241297894. doi: 10.1177/1098612X241297894 (PMC11662329; doi:10.1177/1098612X241297894)
Supplement: sj-docx-2-jfm-10.1177_1098612X241297894 – Supplemental material for Measurement of ground reaction forces in cats after total hip replacement [file sj-docx-2-jfm-10.1177_1098612X241297894.docx]

**File 2 – Technical data of the pressure-sensitive plate**

The gait analysis in Vienna and Ismaning were performed using a Zebris pressure measurement plate:

- Zebris FDM Type 2, Zebris Medical GmbH, Allgäu, Germany
- Dimensions of 203.2 x 54.2 cm, including 15 360 sensors
- Sampling rate of 100Hz.

The plate was placed in the middle of a quiet room and covered with a thin rubber mat to protect against scratches and slipping
