# Supplementary material for: Measurement of ground reaction forces in cats after total hip replacement
Source: J Feline Med Surg. 2024 Dec 20;26(12):1098612X241297894. doi: 10.1177/1098612X241297894 (PMC11662329; doi:10.1177/1098612X241297894)
Supplement: sj-docx-3-jfm-10.1177_1098612X241297894 – Supplemental material for Measurement of ground reaction forces in cats after total hip replacement [file sj-docx-3-jfm-10.1177_1098612X241297894.docx]

**File 3 - Calculation of symmetry index (SI)**

The symmetry index (SI) of the forelimbs and hind limbs was calculated from the PFz and IFz using the following formula:

$$SIXFz (\%)=abs\left( \frac{\left( XFzFL-XFzFR \right)}{\left( XFzFL+ XFzFR \right)} \right)\times100$$

Where the SI = symmetry index, X = the given value of PFz or IFz, FL = front left, and FR = front right.

Hindlimb symmetry was calculated accordingly so that an SI of 0 would represent perfect symmetry between the contralateral limb pair.
