# Supplementary material for: Measurement of ground reaction forces in cats after total hip replacement
Source: J Feline Med Surg. 2024 Dec 20;26(12):1098612X241297894. doi: 10.1177/1098612X241297894 (PMC11662329; doi:10.1177/1098612X241297894)
Supplement: sj-docx-4-jfm-10.1177_1098612X241297894 – Supplemental material for Measurement of ground reaction forces in cats after total hip replacement [file sj-docx-4-jfm-10.1177_1098612X241297894.docx]

**File 4 – Additional results and findings**

**Group-associated findings:**

The mean time between surgery and reexamination was significant longer (p<0.01) in the THR group with 4.4 ± 1.4 (3.1-7.1) years than in the FHO group with 1.9 ± 1.1 (0.8-4.1) years; thus, the THR group was significantly older (p<0.01) than the FHO group at the time of reexamination.

**Orthopedic examination:**

In general, the THR group showed a greater total ROM (p = 0.05) than the FHO group - 119.78 ± 7.77° (THR) vs. 101.56 ± 14.24°.

The hip extension was significantly reduced (p = 0.04) in the FHO group than in the THR group at the operated limb.

A significant correlation was found between lameness and reduced hip extension in the THR group (r = -0.68, p = 0.04).

**Gait analysis:**

A significant correlation was found between decreasing PFz(%TF) of the operated hindlimb and increasing PFz(%TF) and IFz(%TF) for both forelimbs in the FHO group [PFz(%TF) FL-IPS p<0.01 and FL-CL p<0.05, IFz(%TF) both forelimbs p<0.05] and the contralateral forelimb in the THR group (p<0.01 each). A correlation was noted between increasing SI(IFz) of the hindlimbs and increasing IFz(%TF) of the contralateral forelimb in both groups, but only significantly in the FHO group (r = 0.72, p = 0.03). No increase in forces of the contralateral hindlimbs in both groups were seen.

In the FHO group, a significant correlation between increasing TPFz(%SPD) of the operated hindlimb and increasing PFz(%TF) of the contralateral forelimb (r = 0.68, p < 0.05) was noted.

The FHO group showed a correlation between painful hip extension during orthopedic examinations and reduced TPFz(%SPD) scores on the contralateral forelimb (p < 0.05).

**Temporospatial parameters:**

A positive correlation was noted between the age at the time of surgery and increased SPD values on the contralateral forelimb (r = 0.49, p < 0.05).

In both groups, the forelimbs showed larger PCAs than the hindlimbs

**Owner survey:**

A significant correlation was identified between an increased SI(PFz) of the hindlimbs and a poorer evaluation by the owner in the categories jumping on elevations (r = 0.6, p < 0.01), jumping from elevations to the ground (r = 0.61, p < 0.01), and change in gait pattern at the present time (r = 0.53, p < 0.05). In addition, cats after THR with a good owner rating in the category running after lying for a while also showed significantly higher PFz(%TF) values on the operated hindlimb (r = 0.67, p = 0.05).
