# Supplementary material for: Measurement of ground reaction forces in cats after total hip replacement
Source: J Feline Med Surg. 2024 Dec 20;26(12):1098612X241297894. doi: 10.1177/1098612X241297894 (PMC11662329; doi:10.1177/1098612X241297894)
Supplement: sj-docx-5-jfm-10.1177_1098612X241297894 – Supplemental material for Measurement of ground reaction forces in cats after total hip replacement [file sj-docx-5-jfm-10.1177_1098612X241297894.docx]

**File 5 - Lameness scores**

grade 1: slightly disturbed, unclear

grade 2: disturbed, constantly strained

grade 3: disturbed, lameness with compensatory movements

grade 4: disturbed, lameness with compensatory movements with just planar loading

grade 5: disturbed, weight bearing on toes or not at all
