# Supplementary material for: Measurement of ground reaction forces in cats after total hip replacement
Source: J Feline Med Surg. 2024 Dec 20;26(12):1098612X241297894. doi: 10.1177/1098612X241297894 (PMC11662329; doi:10.1177/1098612X241297894)
Supplement: sj-pdf-1-jfm-10.1177_1098612X241297894 – Supplemental material for Measurement of ground reaction forces in cats after total hip replacement [file sj-pdf-1-jfm-10.1177_1098612X241297894.pdf]

Patient: Date:

Pain assessment for cats

Patient

Name:

Breed:

Sex:

Last vet check:

Gets medication: ☐ Yes ☐ No

If yes, which and how often:

Further injuries after hip surgery: ☐ Yes ☐ No

If yes, which and when:

Other diseases after hip surgery: ☐ Yes ☐ No

If yes, which and when:

General behavior

1	2	3	4	5	6	7	8	9	10

Plays a lot, is very curious — *Sleeps a lot, disinterested*

General activity of the cat

1	2	3	4	5	6	7	8	9	10

Moves a lot — *Lies around a lot*

Running

1	2	3	4	5	6	7	8	9	10

No abnormalities — *Severe pain*

Running after lying for a while

1	2	3	4	5	6	7	8	9	10

Patient: Date:

No abnormalities *Severe pain*

Jumping on elevation

1	2	3	4	5	6	7	8	9	10

No problems *Does not jump at all*

Jumping from elevation to the ground

1	2	3	4	5	6	7	8	9	10

No problems *Does not jump at all*

Climbing stairs

1	2	3	4	5	6	7	8	9	10

No problems *will not climbing stairs*

Playing with toys

1	2	3	4	5	6	7	8	9	10

Plays without impairments *No interest*

Pain

1	2	3	4	5	6	7	8	9	10

No pain *Severe Pain*

Gait change at the present

1	2	3	4	5	6	7	8	9	10

No change *Severe lameness*

Appetite

1	2	3	4	5	6	7	8	9	10

Lasting appetite *does not eat at all*
